# Light-Sensitive Open Channel Block of Ionotropic Glutamate Receptors by Quaternary Ammonium Azobenzene Derivatives

**DOI:** 10.3390/ijms241813773

**Published:** 2023-09-07

**Authors:** Maxim Nikolaev, Denis Tikhonov

**Affiliations:** I.M.Sechenov Institute of Evolutionary Physiology and Biochemistry RAS, 194223 St. Petersburg, Russia; denistikhonov2002@yahoo.com

**Keywords:** molecular photoswitch, ion channel, glutamate ionotropic receptor, photopharmacology

## Abstract

Glutamate ionotropic receptors mediate fast excitation processes in the central nervous system of vertebrates and play an important role in synaptic plasticity, learning, and memory. Here, we describe the action of two azobenene-containing compounds, AAQ (acrylamide–azobenzene–quaternary ammonium) and QAQ (quaternary ammonium–azobenzene–quaternary ammonium), which produced rapid and fully reversible light-dependent inhibition of glutamate ionotropic receptors. The compounds demonstrated voltage-dependent inhibition with only minor voltage-independent allosteric action. Calcium-impermeable AMPA receptors had weaker sensitivity compared to NMDA and calcium-permeable AMPA receptors. We further revealed that the compounds bound to NMDA and calcium-permeable AMPA receptors in different modes. They were able to enter the wide selectivity filter of AMPA receptors, and strong negative voltages caused permeation into the cytoplasm. The narrow selectivity filter of the NMDA receptors did not allow the molecules to bypass them; therefore, QAQ and AAQ bound to the shallow channel site and prevented channel closure by a foot-in-the-door mechanism. Computer simulations employing available AMPA and NMDA receptor structures readily reproduced the experimental findings, allowing for the structure-based design of more potent and selective drugs in the future. Thus, our work creates a framework for the development of light-sensitive blockers of calcium-permeable AMPA receptors, which are desirable tools for neuroscience.

## 1. Introduction

Optogenetics and optopharmacology are rapidly developing and powerful tools in neuroscience that allow for selective modulation of the activity of defined populations of neurons with unprecedented specificity [1,2]. An important advantage of optopharmacology is that the activity of freely diffusible compounds can be altered reversibly, locally, and rapidly, with no requirement for genetic manipulations. The further development of these compounds with high activity, selectivity, and a photoswitching effect is an important and largely unresolved problem in neuropharmacology.

Ion channels and ionotropic receptors are among the most important targets of photoswitchable drugs [3,4,5,6]. Ion channels mediate the vast majority of fast components of neural network reactions, which often occur at the level of individual neurons and synapses. For this reason, photoswitchable ligands of ion channels are elaborated upon and studied by many groups. For instance, azocholine provides optical control of α7 nicotinic cholinoreceptors [7], while a propofol-based soluble ligand MPC088 regulates the activity of GABA receptors in a light-dependent manner [8]. DENAQ and related compounds affect various voltage-gated channels [9,10], and diltiasem-based ligands control L-type calcium channels [11].

Ionotropic glutamate receptors mediate fast excitatory synaptic transmission in the central nervous system of vertebrates and regulate a broad spectrum of processes [12]. Among these, the NMDA receptors, which are highly permeable for calcium, are critical for synaptic plasticity. By contrast, the AMPA-type glutamate receptors are split into two categories, one of which is also permeable to calcium (although to a lesser extent than the NMDA receptors), while the other is completely calcium-impermeable. Changes in the subunit composition of AMPA receptors occur during various processes, and an increase in the calcium permeable subtype, in particular, can cause uncontrolled calcium influx and cell death [13]. The list of CNS disorders in which calcium-permeable AMPA receptors are involved includes epilepsy [14], Alzheimer’s and Parkinson’s disorders [15,16], amyotrophic lateral sclerosis [17], glaucoma [18], and hyperalgesia [19]. The overexpression of calcium-permeable receptors during ontogenesis causes abnormal development of the nervous system and fragile X syndrome [20].

Clearly, the identification of selective drugs that can act on particular types of glutamate receptors in a light-dependent manner is desirable in order to study their function, both in health and in disease. There are several examples of freely diffusible photoswitches of glutamate receptors. These include azobenzene-containing derivatives of glutamate (substances ATG [21], Glu-Azo [22]) and its synthetic analog AMPA (substance ATA-3 [23]). The substance Shu-BQX was obtained by modification of competitive antagonists of AMPA receptors [24]. An example of a photochromic NMDA receptor antagonist is the substance PNRA, which shows subtype selectivity for GluN2A and GluN2C over GluN2B and GluN2D [25]. Another attempt to develop a photoswitch selective to the subunit composition was made by modifying ifenprodil, a selective antagonist of the GluN2B-containing NMDA receptor [26]. Currently known photosensitive ion channel blockers of NMDA receptors, azo-memantine and azo-adamantane, demonstrate weak photoswitch effects [25].

The calcium permeability of AMPA receptors is controlled by single residues in the selectivity filter of the ion channel (Q/R site) [12]. Therefore, competitive antagonists and allosteric modulators cannot discriminate between the two types of receptors. By contrast, the action of the pore blockers is highly selective; cationic blockers affect only the calcium-permeable subtype [27], whereas neutral molecules, such as pentobarbital [28,29] and phenytoin [30], preferentially block the calcium-impermeable AMPA receptor channels. The only photoswitchable blocker of calcium-permeable AMPA receptors was obtained by modification of argiotoxin [31]

We recently found that DENAQ and related compounds strongly affect ionotropic glutamate receptors of the NMDA type [32,33]. Unlike its action on voltage-gated channels, which is long-lasting and requires accumulation inside the cells, DENAQ causes a rapid photoswitchable inhibition of NMDA receptors during extracellular application. For some compounds, such as AAQ (acrylamide-azobenzene-quaternary ammonium) and QAQ (quaternary ammonium-azobenzene-quaternary ammonium), a selective effect has been observed on the calcium-permeable, but not on the calcium-impermeable, type of AMPA receptors [33]. Our aim in the present study was to understand the determinants of this selectivity by conducting a detailed investigation of the molecular mechanisms of action of these compounds on glutamate receptors.

## 2. Results

### 2.1. Activity Overview

**Figure 1:**
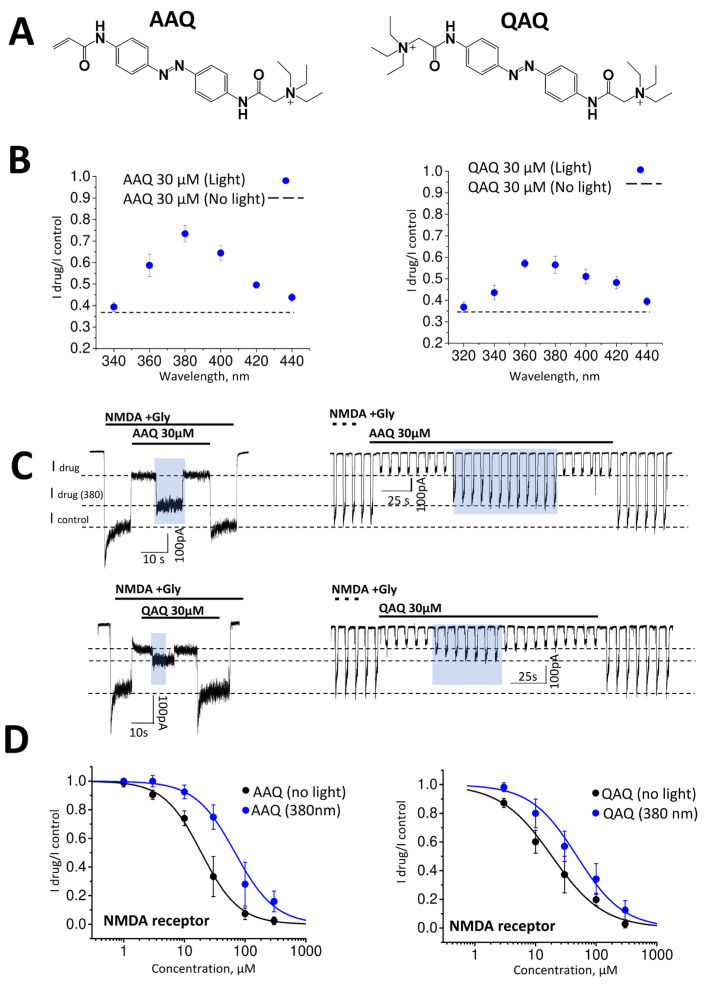
Light-dependent action of AAQ (acrylamide–azobenzene–quaternary ammonium) and QAQ (quaternary ammonium–azobenzene–quaternary ammonium) on NMDA receptors. (**A**) Chemical structures of the compounds. (**B**) Dependence of the effect of AAQ (30 µM) and QAQ (30 µM) on the wavelength of monochromic light (blue symbols). The effect of the drugs under ambient light is shown as dashed lines. (**C**) Representative recording of continuous activation protocol (left, drug effect on the steady-state response) and trains of short activations (right, drug effect on the peak response) are shown. The effects of both compounds in the dark and under illumination (marked with a blue area) in these protocols coincided. (**D**) Concentration dependencies of the blocking effects in the dark and under 380 nm illumination. In all cases, the holding potential was −70 mV.

QAQ and AAQ belong to a family of azobenzene-containing quaternary ammonium derivatives originally developed as photochromic versions of lidocaine [3,5,9]. The chemical structures of the compounds are presented in Figure 1A. We have previously shown that QAQ and AAQ inhibit glutamate receptors at concentrations in the micromolar range [33]. In that study, we used the wavelength corresponding to the absorption maximum (360 nm for both drugs) to induce photoisomerization. Here, we probed the efficiency of the photoswitch effect as a function of the illumination wavelength. The maximum attenuation of the activity of AAQ was observed under illumination with monochromatic light of 380 nm (I drug/Icontrol = 0.73 ± 0.02, n = 6). On the contrary, the effects of the QAQ at 380 nm and 360 nm were statistically indistinguishable (Idrug/Icontrol = 0.57 ± 0.02 at 360 nm and Idrug/Icontrol = 0.56 ± 0.04 at 380 nm; n = 5, *p* = 0.36, paired *t*-test). Thus, we found that for both compounds, the maximum photoswitching effect was observed around 380 nm under our experimental conditions (Figure 1B). Therefore, we used this wavelength for systematic analysis throughout this work.

Next, we compared the effects of extracellularly applied QAQ and AAQ at a concentration of 30 µM on the NMDA type of glutamate receptors using two activation protocols. In a continuous activation protocol (Figure 1C, left), which was used in previous experiments, the agonists (NMDA 100 µM + Gly 10 µM) were applied throughout the recording. In the pulse activation protocol, the agonists were given as short 2 s applications at intervals of 5 s (Figure 1C, right) to measure compound activity in conditions more relevant to synaptic events. This protocol allowed us to measure the drugs’ effects on the peak currents, whereas the protocol with the continuous agonist presence estimated the drugs’ effects on the steady-state response. The pulse protocol that allowed us to monitor the baseline currents also showed no significant changes in the holding currents during drug application without an agonist. The inhibitory effect was completely reversible, and the activities of both compounds coincided in these two protocols (*p* > 0.05, paired *t*-test, Figure 1B). Therefore, in the subsequent experiments, the protocol with continuous agonist presence was used.

The kinetics of the transient processes (the development of inhibition and the photoswitch effect, as well as the recovery after illumination and drugs washout) were fast. We did not quantify these transient processes’ kinetics, since their rates were faster than 200 ms, which lies within the limits of the solution change rate of our application system [34].

Figure 1D shows the complete concentration curves of AAQ and QAQ’s action on NMDA receptors in the dark and under 380 nm illumination. Data obtained using the continuous receptor activation protocol showed that, in the dark, the activities of the compounds were similar (AAQ: IC50 = 19.0 ± 1.4; QAQ: IC50 = 18.6 ± 1.0). The Hill’s coefficient values were also close to 1 in all cases, and the curves shifted in parallel to the right under light conditions. However, the curve shift was greater for AAQ than for QAQ (AAQ: IC50 = 68.4 ± 7.2 at 380 nm; QAQ: IC50 = 48.2 ± 7.7 at 380 nm). Therefore, AAQ demonstrated a better capability for the photocontrol of NMDA receptors.

**Figure 2:**
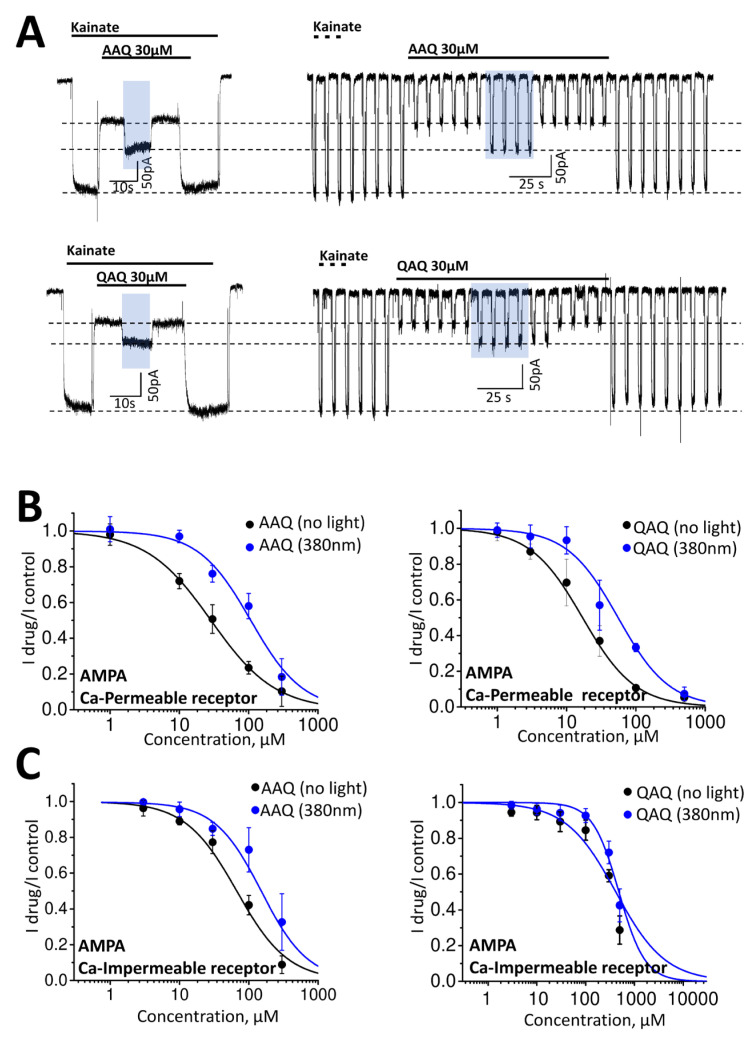
Light-dependent action of AAQ and QAQ on AMPA receptors. (**A**) Representative recording of continuous activation protocol (**left**) and trains of short activations (**right**). The effects of both compounds (30 µM) in the dark and under illumination (marked with a blue area) in these protocols coincided. (**B**,**C**) Concentration dependencies of the blocking effects in the dark and under 380 nm illumination on calcium-permeable (**B**) and calcium-impermeable (**C**) AMPA receptors. In all cases, the holding potential was −70 mV.

The AMPA receptors were also inhibited by QAQ and AAQ. Representative recordings obtained using different activation protocols on calcium-permeable receptors are shown in Figure 2A. As in the case of the NMDA receptors, the level of inhibition and the kinetics were similar with either continuous agonist application or pulsed agonist application. QAQ was slightly more active than its monocationic analog in the dark (AAQ: IC50 = 28.5 ± 1.1; QAQ: IC50 = 17.0 ± 2.0). Illumination evoked an increase in the values (AAQ: IC50 = 107.2 ± 20.6 at 380 nm; QAQ: IC50 = 55.4 ± 1.17 at 380 nm). Interestingly, both substances were less active against the calcium-impermeable AMPA receptors. A concentration of 30 μM caused only ~10–20% inhibition (AAQ: Idrug/Icontrol = 0.77 ± 0.05, n = 5; QAQ: Idrug/Icontrol = 0.89 ± 0.05, n = 7). The maximum concentrations tested were 300 µM for AAQ and 500 µM for QAQ. Complete concentration dependencies could not be determined due to the instability of the registration at high concentrations—a feature that we previously noted for these and some other tetraalkylammonium photoswitches [33].

### 2.2. Voltage Dependence

Both QAQ and AAQ are organic cations that bear +2 and +1 positive charges at physiological pH, respectively. Thus, their action on ion channels should be affected by the electric field across the cell membrane. To test this possibility, we evaluated the activity of AAQ and QAQ on glutamate receptors in a range of membrane holding potentials from +20 to −140 mV.

**Figure 3:**
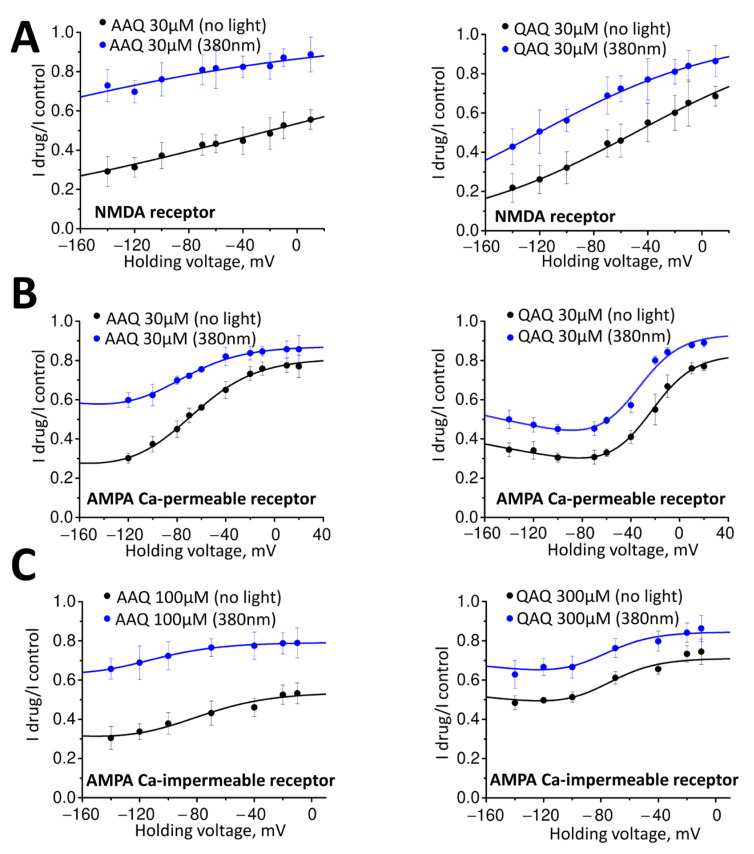
Voltage-dependence of AAQ (**left**) and QAQ (**right**) action on NMDA receptors (**A**); calcium-permeable AMPA receptors (**B**); and calcium-impermeable AMPA receptors (**C**). The action on NMDA receptors is voltage-dependent, whereas a voltage-independent component of action on AMPA receptors is seen at positive voltages. At high negative voltages, saturation of the block or even partial relief is seen for AMPA receptors, but not for NMDA receptors. Experimental data of drugs action on NMDA receptors were fitted by Equation (1), with δ_b_ = 0.18. In the case of AMPA receptors, the experimental data were fitted by Equation (3), with δ_b_ = 0.7 for QAQ and δ_b_ = 0.9 for AAQ; δ_p_ = 0.15 for both compounds.

Figure 3A demonstrates that the inhibition of NMDA receptors by AAQ at 30 µM (left panel) and QAQ at 30 µM (right panel) is voltage-dependent, with a pronounced increase in activity with membrane hyperpolarization (AAQ: I drug/I control = 0.31 ± 0.04 at −120 mV, I drug/I control = 0.46 ± 0.07 at −20 mV, n = 6, *p* = 0.004, paired *t*-test; QAQ: I drug/I control = 0.26 ± 0.06 at −120 mV, I drug/I control = 0.62 ± 0.08 at −20 mV, n = 6, *p* < 0.001, paired *t*-test). The voltage dependence was observed both in the dark and under 380 nm illumination. The experimental data for the voltage dependence were well fitted with the classical model for a charged compound acting as an impermeable ion channel blocker (Equation (1)).

The voltage dependence of doubly charged QAQ was stronger than of AAQ, which is singly charged. The parameter δ_b_, which reflects the relative depth of the binding site in the channel, was similar for both compounds (0.18) under ambient light conditions and under illumination. These results indicate that both the trans- and cis-forms bind to the same site located within the NMDA receptor ion pore, and that the photoswitch effect is due to a decrease in the affinity of the cis-form for this site.

Despite the similar concentration range of activities against NMDA and calcium-permeable AMPA receptors, the voltage dependence of the action of the drugs on calcium-permeable AMPA receptors was markedly different (Figure 3B, right panel). The inhibition did not increase monotonically with hyperpolarization, and a relief from inhibition was observed in the case of QAQ at membrane potentials more negative than −70 mV. This can be explained by the drug permeation inside the cell through the open channel of the AMPA receptors, as demonstrated previously [35,36,37]. Conversely, membrane depolarization led to a residual voltage-independent component of inhibition of 15–20% (Figure 3B). Thus, the voltage-dependence could not be described by Equation (1).

Analysis of this complex voltage-dependence required the introduction of two additional assumptions. First, for the voltage-dependent binding to one of these sites, permeation through the channel should be taken into account (Equation (2)). Second, independent binding to two distinct sites (Equation (3)) should be used.

With these assumptions, the experimental data for QAQ and AAQ were readily fitted. In the case of QAQ, the voltage dependence was reasonably fitted, assuming the parameters of voltage dependence δ_b_ = 0.70 and δ_p_ = 0.15 for both the trans and cis forms. These parameters agreed with previously obtained data for dicationic ion channel blockers [35]. Despite the absence of a clear sign of the penetration phenomenon, the effect of AAQ could also be reasonably fitted with a consensus value of δ_p_ = 0.15. The optimal value of δ_b_ for AAQ was 0.9.

We also studied the voltage dependence of the action of compounds on the calcium-impermeable AMPA receptors. We constructed a complete voltage dependence curve by examining the action of 100 µM AAQ (I drug/I control = 0.43 ± 0.06 at −70 mV, n = 5) and 300 QAQ µM (I drug/I control = 0.61 ± 0.02 at −70 mV). The action of both compounds can be well described using the same values of δ_p_ and δ_b_ as those used for calcium-permeable receptors (Figure 3C). This indicates that these types of receptors have the same binding site within the ion channel.

Thus, our analysis of the voltage dependence of the drugs’ actions suggests that QAQ and AAQ bind to the voltage-dependent site in the NMDA receptor channel. Parameter δ_b_, which reflects the location of the site in the membrane electric field, was much smaller than the value (0.7–0.8) obtained for classical channel blockers, such as memantine [38] and many others. For the calcium-permeable and -impermeable AMPA receptor channels, the parameters of the voltage-dependent block agreed with the previous data obtained for dicationic blockers [35], but additional binding to the voltage-independent site was revealed.

### 2.3. Interaction with the Ion Channel Gates

Pore blockers of glutamate receptors differ according to their interactions with the channel gate. Trapping blockers allow the gates to close and are unable to leave the channel until it opens again. Foot-in-the-door blockers prevent channel closure and must leave the site to allow for channel closure. Classical NMDA receptor channel blockers, such as MK801, ketamine, and phencyclidine, demonstrate a trapping mode [39], whereas the large tetraalkylammonium drugs [40], tacrine [41] and 9-aminoacridine [42] act as foot-in-the-door blockers. Dicationic adamantane derivatives demonstrate two modes of action: in the shallow mode, they prevent channel closure, and in the deep mode, they can be trapped [43].

**Figure 4:**
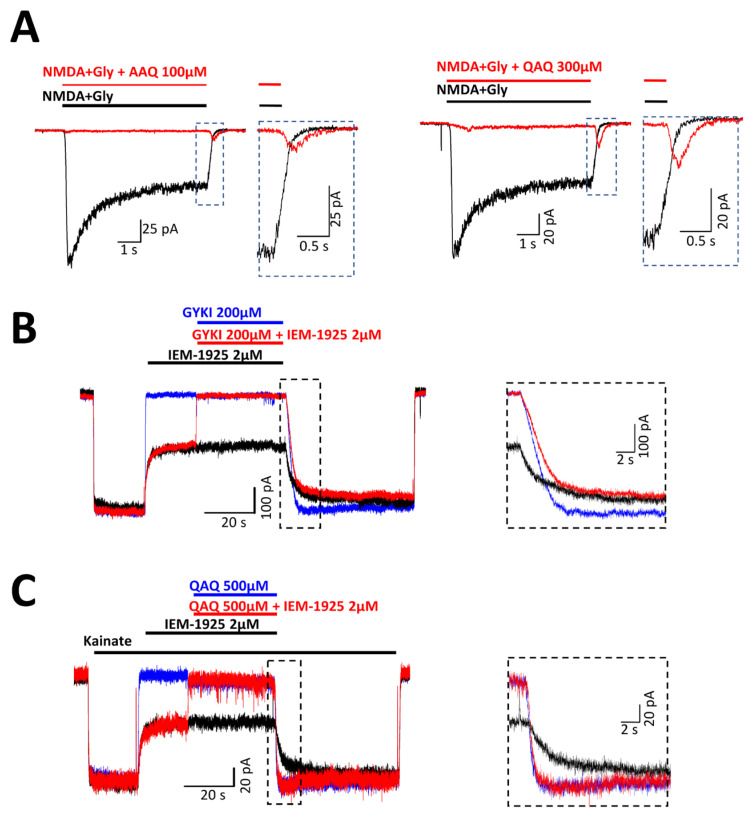
Mechanisms of QAQ and AAQ action on NMDA and AMPA receptors. (**A**) Foot-in-the-door action of AAQ (100 µM) and QAQ (300 µM) on NMDA receptors. Tail currents that prolonged the response after simultaneous washout of a blocker and agonist (red line) suggest that the blocked channels transiently occupy the open state before closure. (**B**) The slow-kinetics AMPA receptor channel blocker IEM-1925 (2 µM, black line) did not compete with a fast-kinetics allosteric modulator GYKI (200 µM, blue line). In the case of a mixture of the two antagonists (red line), the recovery was as slow as in the case of IEM-1925 alone. (**C**) QAQ competed with IEM-1925. In the case of a mixture of the two antagonists, QAQ (500 µM) and IEM-1925 (2 µM), the recovery accelerated, indicating the displacement of IEM-1925 by a high concentration of QAQ (red line). In all cases, the holding potential was −70 mV.

The evidence of an influence of NMDA receptors on channel gating can be found in the protocol when drugs are co-applied with agonists. Termination of the co-application of the agonist and AAQ (100 μM) or QAQ (300 μM) was accompanied by a transient increase in inward current, the so-called tail current. Note that the tail currents were absent in the control when the agonist was applied alone (Figure 4A). Tail currents that prolong the response are a characteristic feature of foot-in-the-door blockers [40]. When this blocker leaves the site, the channels go into a closed state through a transient occupancy of the open state. In our experiments, the tail currents were especially pronounced for QAQ (Figure 4A).

Known blockers of AMPA receptor channels demonstrate a trapping block, but can escape into the cell cytoplasm [35]. The trapping effect is usually revealed by the double-application protocol, in which the channels are first blocked by co-application of the agonist and the blocker. After washout, the second agonist application is used to detect the residual block. Incomplete recovery at the beginning of the second agonist application demonstrated that the blocker was trapped in the channel between the applications. We attempted to employ this protocol for QAQ and AAQ, but the fast kinetics of recovery prevented us from obtaining reliable data.

To overcome this problem, we designed competition experiments using the classical calcium-permeable AMPA receptor ion channel blocker IEM-1925, which demonstrates slow recovery kinetics. QAQ shows very fast kinetics compared to the slow ion channel blocker IEM-1925. The idea of this experiment was that if the QAQ were able to displace IEM-1925 or affect its binding, we would see faster washout kinetics in the protocol when two drugs were applied together than with IEM-1925 alone. If the binding was independent, then the recovery after co-application would remain slow. As a control, we studied the kinetics of IEM-1925 (2 µM) washout in the presence of the fast allosteric antagonist GYKI-52466 (200 µM), which, a priori, had a different binding site. GYKI-52466 was unable to accelerate the response recovery after co-application with IEM-1925 (Figure 4B). Next, we studied the competition between QAQ and IEM-1925 using a saturated concentration of fast QAQ (500 µM), which produced nearly complete inhibition of the receptors. In contrast to GYKI-52466, QAQ markedly accelerated the response recovery, suggesting that it competes with the trapping pore blocker IEM-1925 for the binding site in the pore (Figure 4C).

### 2.4. Modeling the Interactions of QAQ with the NMDA and AMPA Receptor Channels

In contrast to the voltage-independent action, the mechanism and site of which is unknown, the data on the voltage-dependent pore block can be rationalized using structural models. The crystal and cryo-EM structures of NMDA and AMPA receptor channels are available and can be used for ligand docking. For our modeling, we used the 6wht [44] and 6cna [45] structures of the NMDA receptor channel, the 6qkc [46] structure of the calcium-impermeable AMPA receptor channel, and the 6dm0 [47] structure of the calcium-permeable AMPA receptor channel. The pore-forming domain models of these structures, which included M1, M2, and M3 segments, were initially optimized using the MCM protocol to obtain energetically favorable structures. The overall similarity with the initial structures was maintained by “pin” constraints, which allow the alpha carbons to freely deviate from their positions in the experimental structures by 1 Å and impose a parabolic energy penalty for larger deviations. For all models, the energetically optimal structures calculated by MCM protocol did not incur any energy penalty from the pin constraints used.

**Figure 5:**
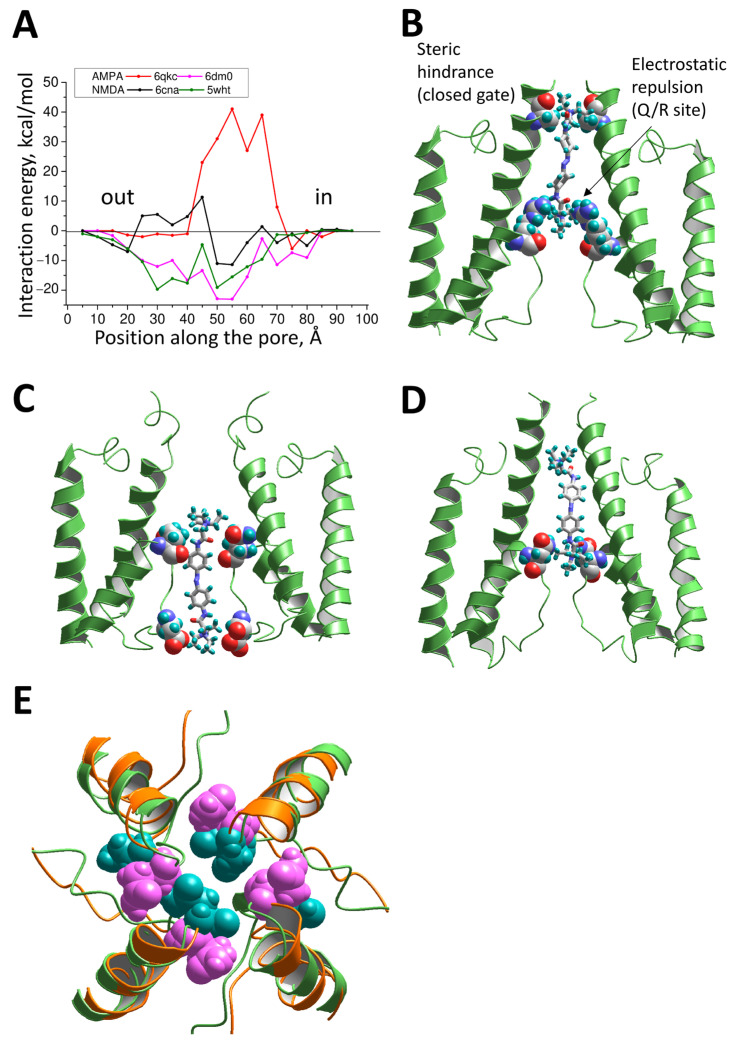
Binding of QAQ in the trans conformation to the NMDA and AMPA receptor channels. (**A**) Energy profiles of QAQ’s movement through the channel models. A high-energy barrier for the 6qkc structure is provided by the closed channel gate and by the arginines of the selectivity filter. Hindrances from the closed gate are also seen in the energy profile for the 6cna structure. The open-gate AMPA receptor structure 6dm0 gives a smooth profile with a wide minimum. (**B**–**D**), Representative structures for the 6qkc (**B**), 6dm0 (**C**), and 6wht (**D**) structures. (**E**) Comparison of the selectivity filter dimensions in the 6wht (green backbone, cyan atoms) and 6dm0 (orange backbone, magenta atoms) structures. The selectivity filter is narrower for the NMDA receptor (6wht) than for the AMPA receptor (6dm0). This difference determines the different binding modes of QAQ in the NMDA and AMAP receptor channels (**C**,**D**).

We analyzed the QAQ binding by calculating the energy profiles. The QAQ molecule was placed above the channel at the pore axis in an axial orientation, and a series of MCM optimizations was performed with a systematic shift of the initial QAQ position along the pore at 5 Å steps. At each position, the energy was calculated for the QAQ interactions with the channel in the best-energy structure. The results are shown in Figure 5. The profile obtained for the 6qkc structure has a large and wide maximum that corresponds to repulsive interactions. Inspection of the contributions of particular residues to this energy maximum demonstrated that the left (external) part is formed by the alanine residues in the closed-channel bundle at the SYTANLAAF motif (Figure 5B), while the internal part formed by electrostatic repulsion with Arg residues at the Q/R site. Thus, our calculations confirmed that a channel block of the calcium-impermeable AMPA receptors by QAQ is impossible. Another conclusion is that closed gates prevent entry of the drug into the pore.

Drastically different energy profiles were obtained for the open-gate structure of the calcium-permeable AMPA receptor 6dm0. A deep and wide energy minimum was seen in the energy profile. The external part corresponded to the QAQ location above the selectivity filter. In the most intracellular part of the minimum, the molecule penetrated the selectivity filter, and only the headgroup was located in the external vestibule (Figure 5C). This binding mode agreed with the position of IEM-1460 in the experimental 6dm0 structure [47]. Glutamine residues in the selectivity filter contributed to the interaction energy in both cases. Multiple M3 residues provided additional stabilization of the complex if the QAQ was in the shallow position. Selectivity filter residues downstream from the Q/R site (particularly the aspartate at position +3, see Figure 5C) interacted with the QAQ in the deep binding mode.

The energy profiles for the NMDA receptor structures 6can and 6wht were drastically different in the left (external) part. The closed-gate structure 6cna demonstrates a clear energy barrier, which is absent in the open-gate structure 6wht. However, in 6wht structure, the region of negative energies is separated into two parts by a local maximum at position 45. In this position, the headgroup of QAQ entered the selectivity filter and sensed steric repulsion (Figure 5D). This type of local maximum was absent in the AMPA receptor channel structure because of the wider dimensions of this channel, which was obvious when comparing the 5wht and 6dm0 structures (Figure 5E). This difference in the selectivity filter lumen agrees with experimental data on the permeability for organic ions [48,49]. Thus, QAQ is unlikely to bypass the narrow selectivity filter of the NMDA receptor channels, and can, therefore, bind only in the shallow binding mode. By contrast, in the calcium-permeable AMPA receptor channel, QAQ can bind in the deep mode.

The comparison of the proposed binding modes shown in Figure 5C,D demonstrates good agreement with the voltage-dependence data. In the NMDA receptor channel, QAQ binds in the shallow mode above the selectivity filter, while in the AMPA receptor, it binds in the deep mode. The δ values obtained for QAQ in our experiments on the AMPA and NMDA receptors fully agreed with these model predictions. The experimental data on the trapping mode in the AMPA receptor and the foot-in-the-door mode in the NMDA receptor were also in agreement with the predictions of the models. In the deep mode, the QAQ molecule did not prevent channel closure, since only a headgroup occupied the cavity. However, in the shallow binding mode, the long QAQ molecule did not allow the channel to close, and therefore corresponded to the foot-in-the door block.

**Figure 6:**
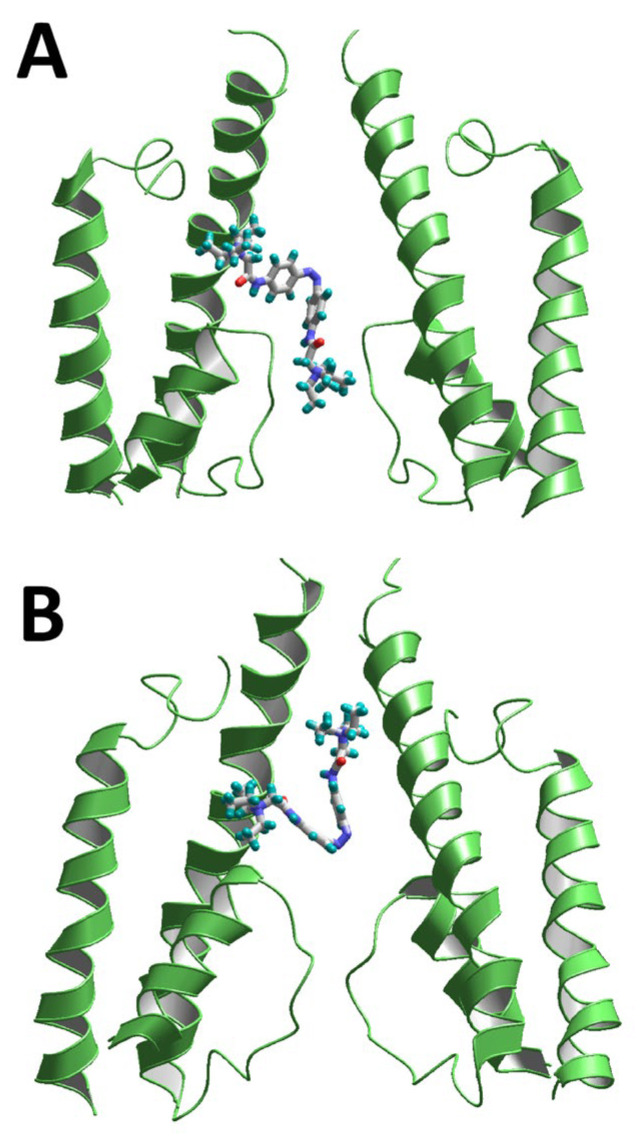
Predicted binding of QAQ in the *cis* conformation to the AMPA (6dm0, **A**) and NMDA (6wht, **B**) receptor channels. One terminal moiety of QAQ penetrates into the subunit interface in both channels.

All the previous calculations were performed without conformational restrictions on the QAQ molecule, and it always was in the energetically optimal trans form. We also evaluated how the cis form of the molecule could bind in the channels by imposing an additional constraint that maintained the QAQ molecule in its cis conformation. The result of the docking is shown in Figure 6.

The change in the overall shape of the QAQ molecule in the cis conformation resulted in alternation of the binding modes in the NMDA and AMPA receptor channels. One end of the molecule enters the subunit interface above the selectivity filter. In the homologous voltage-gated calcium and sodium channels, this region forms a classical binding site for various ligands. Even in this angular conformation, QAQ can bind in both channels with negative (attractive) energies. For the NMDA and AMPA receptor channels, the QAQ interaction energies were −7.2 and −11.6 kcal/mol, respectively. This is why illumination caused a significant change in QAQ and AAQ activity, but did not completely prevent binding. In the NMDA receptor channel, the subunit interface provides an access route for memantine [50]. For the AMPA receptor channel, the binding mode in the subunit interface was previously proposed for fluoxetine [51]. The possible binding of QAQ in its cis form explains the moderate photoswitch effect observed in our experiments. Thus, our modeling results provide structural rationalization for the key experimental findings regarding the voltage-dependent QAQ action on the glutamate receptor channels.

## 3. Discussion

In recent papers, we have demonstrated that relatively simple azobenzene-containing amines are able to modulate glutamate ionotropic receptors in a light-dependent manner [32,33]. These drugs show differential selectivity to the glutamate receptor types, and their modulatory action may be due to interactions with different parts of the receptors. For example, DENAQ (diethylamine–azobenzene–quaternary ammonium) and its close analogue PyrAQ (pyrrolidine–azobenzene–quaternary ammonium) are selective light-dependent antagonists of NMDA receptors. PyrAQ inhibits the NMDA receptors regardless of agonist concentration, receptor activity, or membrane potential, and it most probably acts as an allosteric modulator. We also found that QAQ and AAQ inhibit both NMDA and AMPA receptors. In the present work, we studied the action of QAQ and AAQ in detail.

Our principal finding is that these compounds, unlike DENAQ and PyrAQ, act as voltage-dependent open-channel blockers. Only a minor component of action on AMPA receptors can be explained as voltage-independent allosteric inhibition. All characteristics of the action are in agreement with the classical views of blockage of the pore of an ionotropic glutamate receptor by organic cations. The calcium-impermeable AMPA receptors are weakly sensitive to these cationic compounds because of the arginine residues in their selectivity filter.

The voltage dependence of the channel block is stronger for calcium-permeable AMPA receptors than for NMDA receptors. The origin of this difference is the well-known difference in the lumen dimensions at the level of selectivity filters: the AMPA receptor channels are significantly wider than NMDA receptor channels. As a result, QAQ and AAQ can penetrate the selectivity filter of AMPA receptors and bind deeply. This binding mode has been experimentally revealed for other AMPA receptor channel blockers and is visualized in structural studies. Moreover, strong negative voltages push the blocking molecules through the channel, thereby providing partial relief from block. By contrast, the selectivity filter of the NMDA receptors is too narrow to allow for QAQ or AAQ penetration. In this shallow mode, they prevent channel closure in the same fashion as other large-size dicationic compounds do [43]. All these features of the block were readily reproduced by computational experiments with the available atomic-scale structures of NMDA and AMPA receptors.

Although QAQ and AAQ poorly discriminate NMDA and calcium-permeable AMPA receptors, our results strongly suggest that they bind to non-identical sites. This finding creates the possibility of designing selective compounds in the future. Allosteric inhibitors and competitive inhibitors are unable to resolve this pharmacological problem since calcium-permeable and calcium-impermeable receptors differ mainly in their selectivity filters (Q/R site). The development of light-sensitive selective pore blockers of calcium-permeable AMPA receptors will provide neurophysiologists with a desirable tool that can control this specific and important subtype of glutamate receptors.

## 4. Materials and Methods

### 4.1. Electrophysiology

Wistar rats (14–21 old; both sexes) were obtained from the animal facility at the Sechenov Institute of Evolutionary Physiology and Biochemistry of the Russian Academy of Sciences (IEPhB RAS). Experiments were performed in accordance with European Directive 2010/63/EU and were approved by the Local Bioethics Committee of the IEPhB RAS (protocol #1-19/2023). The rats were anesthetized with isoflurane, sacrificed by cervical dislocation, and then decapitated. The brains were removed and immersed in ice-cold (2–4 °C) artificial cerebrospinal fluid (ACSF) with the following composition (in mM): NaCl, 124; 5 KCl, 5; CaCl_2_, 1.3; MgCl_2_, 2; NaHCO_3_, 26; NaH_2_PO_4_, 1.24; and D-glucose, 10. The brains were cut with a vibratome (7000 SMZ-2, Campden Instruments, Leicestershire, GB) into slices comprising the hippocampus and striatum, which were then stored in carbogen-aerated ACSF (95% O_2_, 5%, CO_2_; 22–24 °C).

The effects of the drugs were tested on the hippocampal pyramidal neurons of the CA1 area, expressing the NMDA and AMPA calcium-impermeable receptors [52,53,54,55], and on the giant cholinergic interneurons of the striatum, expressing calcium-permeable AMPA receptors [56]. The expression of calcium-permeable AMPA receptors in hippocampal pyramidal cells of the CA1 region cannot be entirely excluded, especially regarding the age of the animals used in this study [56,57,58]. We regularly used IEM-1925, a voltage-dependent and use-dependent channel blocker of calcium-permeable AMPA receptors, as a reference compound for the pharmacological identification of a receptor subpopulation (Tikhonov et al., 2000) [37]. The kainate-induced currents in hippocampal CA1 pyramidal neurons were insensitive to 1 µM IEM-1925, confirming the virtual absence of calcium-permeable AMPA receptors and their negligible contribution to the observed effect of the photoswitches. On the contrary, deep inhibition of the AMPA responses in giant striatal interneurons by 1 µM IEM-1925 (about 50%) indicated that the subpopulation of calcium-permeable receptors dominated in these cells.

Neurons were isolated from the slices by a vibrodissociation method without enzymatic treatment [59]. The extracellular solution contained (in mM): NaCl (143), KCl (5), CaCl_2_ (2.5), D-glucose (10), and HEPES (10); the pH was adjusted to 7.4 with HCl at 22–24 °C. Whole-cell currents through glutamate receptors were recorded using an EPC-10 patch clamp amplifier (HEKA Elektronik, Germany) in the voltage clamp mode. Receptors were activated by the application of specific agonists (NMDA receptor: NMDA 100 µM + glycine 10 µM; AMPA receptors: kainate 100 µM). Signals were filtered at 5 kHz and sampled at 20 kHz. The series resistance of about 20 MΩ was compensated by 70–80% and continuously monitored during the experiments; it remained stable (≤20% increase) in all cells included in the analysis.

QAQ and AAQ, as well as all drugs for the ACSF and intracellular pipette solutions, were purchased from Tocris. The drugs were applied using an RSC-200 fast perfusion system (BioLogic, France). The patch pipettes were made of borosilicate glass (WPI). The intrapipette solution contained (in mM): CsF (100), CsCl (40), NaCl (5), CaCl_2_ (0.5), EGTA (5), and HEPES (10); the pH was adjusted to 7.2 with CsOH.

We used a monochromator (Optoscan, Cairn, UK) equipped with a 150 W xenon arc lamp as a source of monochromatic light. The light was focused onto the isolated neuron with a 40× objective (MRH10401, Nikon, Tokyo, Japan).

The ratio of the current in the presence of the drug to the current in the control condition (I drug/I control) was calculated in order to characterize the effect of the drugs. All data are presented as means ± SD, estimated from at least five experiments; n = number of neurons.

The significance of the effects was evaluated by means of a two-tailed Student’s paired *t* test. Data obtained from two groups of different cells were compared using an unpaired Student’s *t* test. In all cases, a value of *p* < 0.05 was considered statistically significant. The normality of data distribution was verified using the Shapiro−Wilk test. The data were analyzed using Origin 9.1 (OriginLab Corp., Northampton, MA, USA) software.

### 4.2. Analysis of the Voltage-Dependence

In the case of a voltage-dependent pore block, the effect of the drug (I drug/I control) at concentration C and membrane voltage V is given by the equation:

Idrug/Icontrol = 1/(1 + C/K_b_ exp(Fzδ_b_V/R/T)) (1)

where K_b_ is affinity of a drug to the channel and δ_b_ is “electrical depth” of the binding site, and F, z, R, and T are Faraday’s constant, molecular charge, gas constant, and temperature, respectively. Taking into account the blocker molecule permeation through the channel results in the following equation:

Idrug/Icontrol = 1/(1 + C/(K_b_ exp(Fzδ_b_V/R/T) + K_p_ exp(−Fzδ_p_V/R/T))), (2)

In this equation, K_b_ and δ_b_ characterize binding to the site, whereas K_p_ and δ_p_ describe permeation [35]. To characterize independent binding to two distinct binding sites, this equation should be combined with the following:

Idrug/Icontrol = 1/(1 + C/(K_b_ exp(Fzδ_b_V/R/T) + K_p_ exp(−Fzδ_p_V/R/T)) + C/K_vin_ + C^2^/((K_b_ exp(Fzδ_b_V/R/T) + K_p_ exp(−Fzδ_p_V/R/T)) K_vin_)) (3)

where K_vin_ is affinity of the drug to the voltage-independent site.

### 4.3. Molecular Modeling

Molecular modeling was performed using the ZMM program package [60]. The nonbonded energy was calculated using the AMBER force field [61], and the hydration energy was calculated using the implicit solvent method [62]. Electrostatic interactions were calculated using the distance-dependent dielectric function, and the atomic charges of diamidine compounds were calculated using the semiempirical method AM1 [63]. The Monte Carlo with energy minimizations method [64] was used to optimize the models and their complexes with the drugs. During energy minimizations, the alpha carbons of the P-helices were constrained to the corresponding positions of the template. The models were optimized until 1000 consecutive minimizations did not decrease the energy of the apparent global minimum.

## Data Availability

The authors confirm that the data supporting the findings of this study are available within the article. They are also available from the corresponding author upon reasonable request.
